# Synthesis of Canthardin Sulfanilamides and Their Acid Anhydride Analogues via a Ring-Opening Reaction of Activated Aziridines and Their Associated Pharmacological Effects

**DOI:** 10.3390/molecules21010100

**Published:** 2016-01-16

**Authors:** Ling-Ling Chiang, Ing-Jy Tseng, Pen-Yuan Lin, Shiow-Yunn Sheu, Ching-Tung Lin, Yun-Han Hsieh, Yi-Jing Lin, Hsiao-Ling Chen, Mei-Hsiang Lin

**Affiliations:** 1 School of Respiratory Therapy, Taipei Medical University, Taipei 11031, Taiwan; llchiang@tmu.edu.tw; 2 Chest Medicine Department, Shuang-Ho Hospital, Taipei Medical University, Taipei 11031, Taiwan; 3 Gerontology Health Management, College of Nursing, Taipei Medical University, Taipei 11031, Taiwan; Ingjy@tmu.edu.tw; 4 Department of Pharmaceutical Sciences, School of Pharmacy, Taipei Medical University, Taipei 11031, Taiwan; lpy0620@tmu.edu.tw (P.-Y.L.); amel@tmu.edu.tw (S.-Y.S.); jay4273803@hotmail.com (Y.-H.H.); m301101003@tmu.edu.tw (Y.-J.L.); m301101001@tmu.edu.tw (H.-L.C.); 5 Department of Chemistry, Tam-Kang University, Danshui 25137, New Taipei City, Taiwan; rocketqueenohya@hotmail.com

**Keywords:** cantharidin, cantharidinimide, methylsulfanylaziridine

## Abstract

The cantharidinimide derivatives, **5a**–**h**, including sulfanilamides containing pyrimidyl, pyrazinyl, hydrogen, thiazolyl, and oxazolyl groups were synthesized. Modification of cantharidinimide by means of the reaction of activated aziridine ring opening led to the discovery of a novel class of antitumor compounds. The analogues **10i**–**k**, **11l**–**n**, **12o**–**p**, and **16q**–**s** were obtained from treating cantharidinimide **6** and analogues (**7**, **8**, and **13**) with activated aziridines, which produced a series of ring-opened products including normal and abnormal types. Some of these compounds showed cytotoxic effects *in vitro* against HL-60, Hep3B, MCF7, and MDA-MB-231 cancer cells. The most potent cytostatic compound, *N*-cantharidinimido-sulfamethazine (**5a**), exhibited anti-HL-60 and anti-Hep3B cell activities. Two compounds **5g** and **5h** displayed slight effects on the Hep3B cell line, while the other compounds produced no response in these four cell lines.

## 1. Introduction

**Chart 1:**
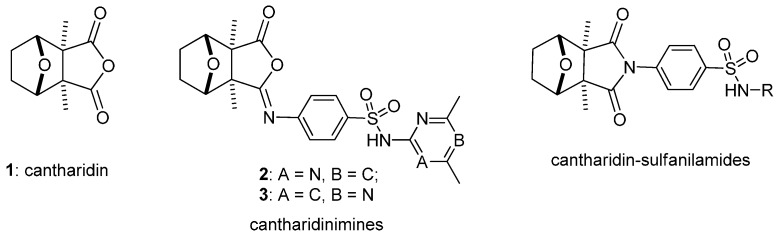
Structures of cantharidin (**1**), cantharidinimines (**2**, **3**), and cantharidin-sulfanilamides.

Cantharidin **1** (Chart 1), isolated from *Mylabris caraganae* and various other insects, is a terpenoid and has a high vesicant potency [1]. It is used to treat piles, warts, ulcers, and molluscum through topical application and is also used as an abortifacient and aphrodisiac [1,2]. In clinical studies, it had significant activity against liver tumors and the KB cell line in tissue culture at low concentrations [3,4]. The clinical application of cantharidin is limited by its cytotoxic properties [5,6]. It is an inhibitor of the serine/threonine protein phosphatases, PP1 and PP2A, leading to multiple cellular effects such as DNA damage, cell cycle arrest, and apoptosis [7,8,9,10]. Cantharidin induces apoptosis in many human cancer cells such as pancreatic carcinoma [9], colon cancer [10], lung cancer [11], melanomas [12], and bladder cancer [13]. It also induces endoplasmic reticular (ER) stress and autophagic cell death [13,14,15]. Recently, certain reports indicated that *N*-cantharidin derivatives have antitumor and antihepatoma properties [16,17,18,19]. Some cantharidinimides showed tumor-inhibitory action in animals, and several cantharidinimides exhibited inhibitory effects against xanthine oxidase [20]. A number of modified cantharidin analogues were synthesized and their anticancer activities against a wide range of human tumor cell lines were evaluated [21,22,23,24,25,26,27].

In our previous research, we synthesized many cantharidinimides by modification with alkyl, phenyl, pyridinyl, thiazolyl, and thiadiazolyl groups [28,29,30]. Those compounds showed certain degrees of cytotoxic activities against HL-60 (human myeloid leukemia cells), Hep 3B and Hep G2 (human hepatocarcinoma cells), NUGC (human gastric carcinoma cells), and HONE-1 (human nasopharyngeal carcinoma cells). Cantharidinimides with better solubility showed greater cytotoxicity against human hepatoma cell lines. The cantharidinimides with an electron-withdrawing group (R_1_) on the aromatic ring (X) exhibited increased cytotoxicity to cancer cell lines. Then, we used this feature and synthesized different imines to study their cytotoxic effects [31]. These results showed that cantharidinimine derivatives had greater cytotoxic effects than cantharidinimides. Especially, cantharidinimine derivative **2** (Chart 1), which has a sulfanyl group, exhibited a 50% inhibitory concentration (IC_50_) value of 6 µM against HL-60 cells as did cantharidin (IC_50_ value of 7.2 µM).

However, cantharidinimides and their analogues also display inhibition of the inducible nitric oxide (NO) synthase (iNOS) pathway, and they inhibited NO synthase activity by more than 90%. Thus, they might be advantageous for anticancer therapy [32,33]. Because of this, we synthesized canthardin sulfanilamides (Chart 1) and tested their cytotoxic effects on human cancer cell lines (HL-60 and Hep3B), human breast carcinoma cells (MCF7), and human breast carcinoma cells (MDA-MB-231) since incidence rates occur rapidly and more widely among Asian peoples than in Westerners. Growth inhibition was evaluated using an MTT assay. In order to find new cantharidinimides and related imides containing the sulfonamide group, we synthesized sulfonamido-ethyldicarboximide derivatives (**10i**–**k**, **11l**–**n**, **12o**–**p**, and **16q**–**s**) and tested their cytotoxic activities.

## 2. Results and Discussion

### 2.1. Chemistry

**Scheme 1:**
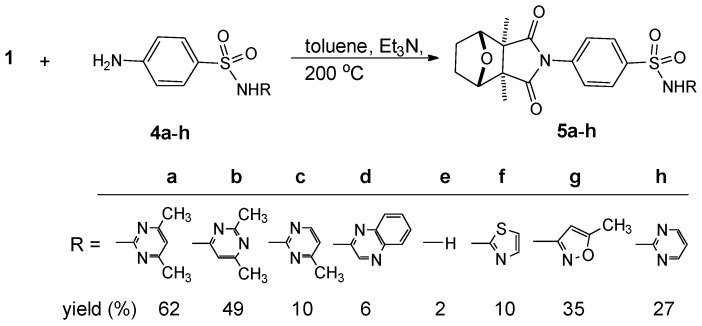
Synthesis of cantharidin-sulfanilamides **5a**–**h**.

We prepared effective cantharidin sulfanilamides by heating the reactant cantharidin (**1**) and primary arylamines of sulfanilamide derivatives (**4a**–**h**) to *ca.* 200 °C with 3 mL of dry toluene and 1–2 mL of TEA in a high-pressure sealed tube (Buchi glasuster 0032) to provide compounds **5a**–**h** in good yields (Scheme 1). The sulfonamide derivatives containing pyrimidine, pyrazine, hydrogen, thiazole, and oxazole were sulfadiazine, sulfamethazine, sulfisomidine, sulfamerazine, sulfaquinoxaline, sulfacarbamide, 2-thiazolyl-sulfanilamide, and sulfamethoxazole (**4a**–**h**). Yields varied 2%–62% and showed a trend compatible with the expected basicity, and the characters of the bulky quinoxaline and the more-basic carbamide groups influenced compounds **5d** (6% yield) and **5e** (2% yield). The results obtained with **5a**–**c** and **5f**–**h**, however, showed the same strong electron-withdrawing sulfonyl (SO_2_) group and aromatic amino (NH_2_) basicity, which were unknown but really influenced the product yields, and the characters of a long side chain with resonance and induction effects were considered spontaneously.

**Scheme 2:**
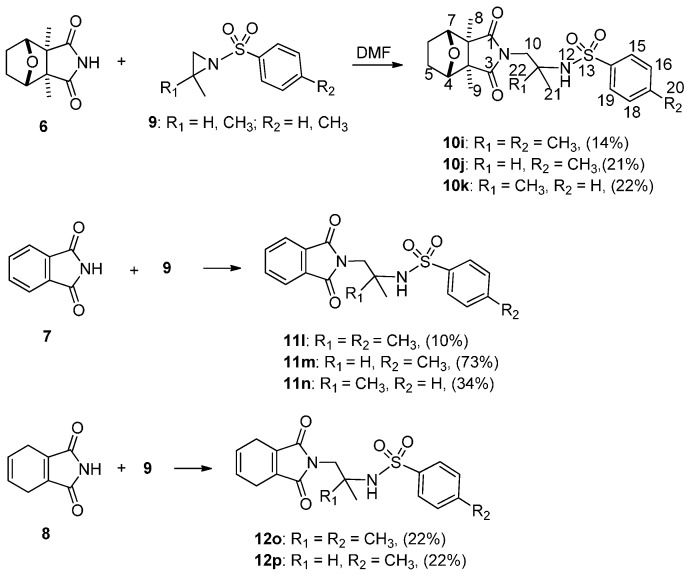
Synthesis of compounds **10i**–**k**, **11l**–**n**, and **12o**–**p**.

**Figure 1:**
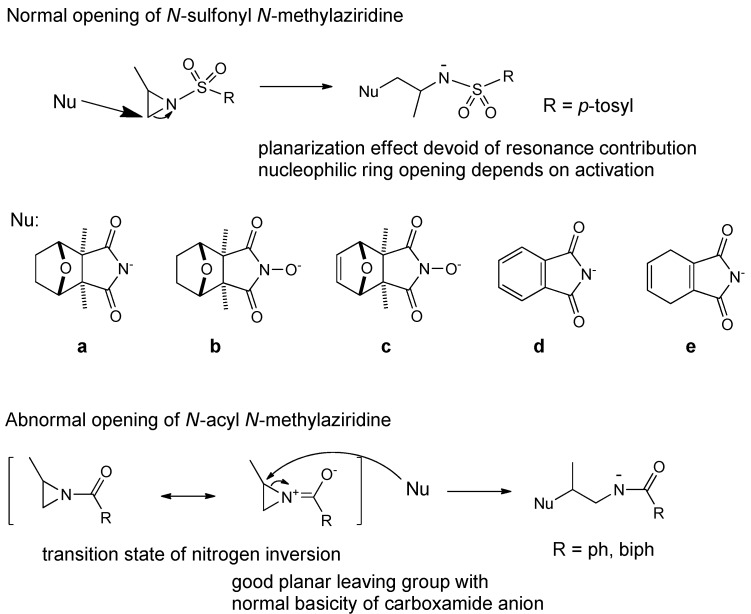
Regioselectivity in nucleophilic ring opening of *N*-sulfony and *N*-acyl activated 2-methylaziridine.

In order to find some new cantharidinimides, cantharidin was treated with ammonia and yielded cantharidinimide (**6**) which was reacted with dimethyl or monomethyl-activated aziridins. Thus, **6** and its imide derivatives (**7** and **8**) were reacted with *N,N’*-dimethylformamide (DMF) in a sealed tube under reflux for 3 h, after which it was cooled, and then activated aziridine (**9**) was added dropwise and refluxed for another 2 h to yield compounds **10i**–**k**, **11l**–**n**, and **12o**–**p** (Scheme 2). In this study, the ring opening reaction of *N*-sulfonyl 2,2-dimethylaziridine by imide nucleophiles (Figure 1a,d,e), which attacked the unsubstituted carbon, was detected. However, the ring-opening reaction of *N*-acyl 2,2-dimethylaziridine by various nucleophiles in the absence of an acid was reported to proceed with a formal attack on the tertiary carbon of the aziridine ring (abnormal opening) [34]. No products of a “normal opening” (in the S_N_2 sense) were observed when the R groups were phenyl or biphenyl (Figure 1). The small amount of normal opening yield (when R was phenyl) was possibly the result of a displacement in the inversional ground states. It was through the regioselectivity ring-opening reaction, and the strong preference for normal-opening sulfonyl activation (R group was tosyl) that simply reflected the steric hindrance of a nucleophilic attack on the tertiary aziridine carbon in the inversional ground state. A single electron transfer (SET) mechanism was proposed to account for this unexpected behavior [35,36]. Therefore, we proposed that both normal and abnormal opening reactions of activated aziridines were slowed down compared to a reaction in the absence of the two methyl groups, the former clearly more so than the latter, and the white crystalline compounds **10i**–**k** were obtained (Scheme 2). The same method was used with cantharidin analogue compounds to obtain products **11l**–**n** and **12o**–**p**.

**Figure 2:**
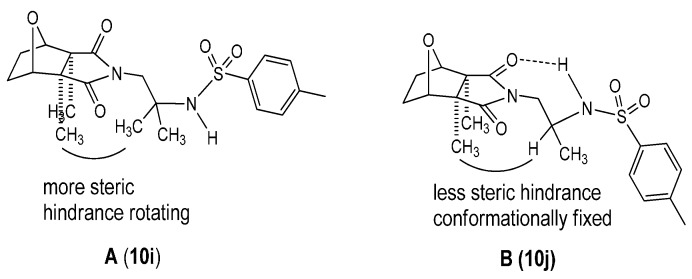
Conformation of **10i** and **10j**.

Structures of products **10j** and **10i** followed from the ^1^H-NMR spectra of chromatographic fractions, and the main evidence for **A** included signals of cantharidinimide’s CH_3_ (position 8 and 9), NCH_2_ (position 10), and OCH (position 4 and 7) protons at 1.22 (singlet), 3.51 (singlet), and 4.54 ppm (singlet), respectively (Table 1, Figure 2). These signals indicated the same values of the protons on two kinds of methyl, OCH, and NCH_2_ groups of product **10i** which might have greater steric hindrance (compared to **10j**, Figure 2B) repulsion between the two pairs of methyl groups, and the conformation for **10i** might have the least electrostatic repulsion between the proton of nitrogen and the two oxygen atoms of cantharidinimide with their negative partial charge. A downfield shift of product **10j** relative to **10i** was observed for these protons. These shifts and coupling constants (Table 1) became complicated for several possible reasons: the hydrogen bonding bridge enhanced the electron-withdrawing capacity of the sulfonyl group and shifted the signals downfield from the usual range, and the three methyl groups might have less steric hindrance to each other. Thus, the structure became the conformationally fixed type B (Figure 2).

**Table 1:** ^1^H chemical shifts (*™*) of sulfonamide-cantharidinimides **10i** and **10j** (CDCl_3_, 500 MHz).

Position	10i	10j
	δ_H_ (*J* in Hz)	δ_H_ (*J* in Hz)
4, 7	4.54 (s)	4.55 (s)
5, 6	1.68–1.70 (m); 1.79–1.81 (m)	1.67-1.70 (m); 1.79–1.83 (m)
8, 9	1.22 (s)	1.21 (s)
10	3.51 (s)	3.38 (dd, 3.8, 3.8); 3.61–3.64 (m)
11	-	3.52 (dd, 9.7, 9.7)
12	5.77 (s)	4.68 (d, 9)
15, 19	7.72 (d, 8)	7.70 (d, 8)
16, 18	7.23 (d, 8)	7.27 (d, 8)
20	2.39 (s)	2.41 (s)
21	1.14 (s)	0.93 (d, 6.7)
22	1.14 (s)	-

**Scheme 3:**
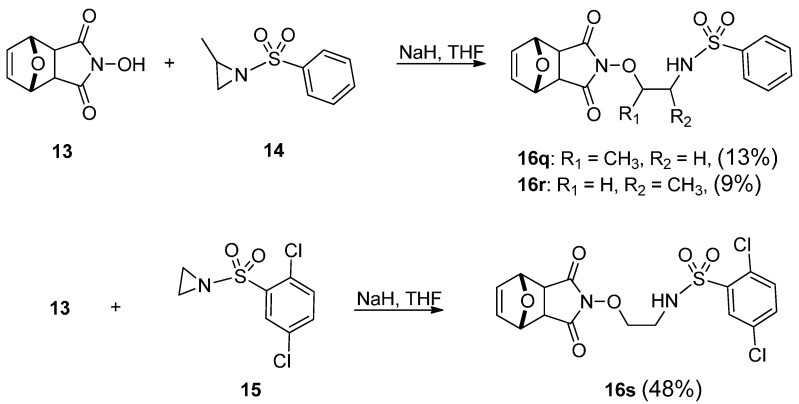
Synthesis of compounds **16q**–**s**.

The *N*-pyrimidyl, *N*-thiazolyl, *N*-oxazolylsulfonylcantharidinimides, **5a**–**h**, were prepared by means of a pressure technique synthesis, and their analogous derivatives (**10i**–**k**, **11l**–**m**, and **12o**–**p**) were synthesized via nucleophilic ring opening of methyl- or dimethylbenzenesulfonylaziridine and methyl- or dimethyltoluenesulfonylaziridine, after reacting with several analogous nucleophilic bases. Compounds **16q**–**s** were obtained from *N*-hydroxylimide **13** and activated aziridine by treatment with NaH base in dry THF for 24 h (Scheme 3). After evaporation, it was purified by silica gel column chromatography and recrystallized in methanol. From these reactions, it should be noted that the characters of various bases (Figure 1a–e) strongly confirmed the influence of the positions of the ring opening of the activated aziridines. The preparative technique, the temperature, the drying solvent, and the formation of nucleophilic bases were also influenced by other factors that can cause strong variations in the results. The reaction steps and time were also crucial factors in this formation. The results of these yields strongly confirmed the influence of the sulfanilamide side chain (**5e**, 2%, R = H).

### 2.2. Pharmacology

Cytotoxicities of the cantharidin-sulfanilamide derivatives, **5a**–**h**, were tested by suppressing the growth of the HL-60, Hep3B, MCF7, and MDA-MB-231 human carcinoma cells using MTT cell viability assays. Some compounds showed inhibitory effects on HL-60 and Hep3B cells. Cantharidin **1** was more toxic and exhibited greater cytotoxicity, while the synthesized cantharidinimides were less toxic but also exhibited inhibitory effects (Table 2). In this study, compound **5a**, which had two methyl groups on the pyrimidyl ring, displayed strong inhibitory effects on the HL-60 and Hep3B cell lines, as did cantharidin. *N*-Cantharidinimido-sulfamethazine **5a** was less toxic compared to *N*-cantharidinimino-sulfamethazine **2** against HL-60. This result confirmed our previous study. Compounds **5b** and **5c** also had methyl groups, but, unfortunately, no activities were detected against either of the two cell lines as compounds **5h** and **5d**, thus the methyl group positions highly influenced the results. Compound **5g** with an oxazolyl group exhibited medium cytotoxicity against the Hep3B cell line, but compound **5f**, although with a thiazolyl group, displayed no inhibitory effects on either cell line. Aziridine side chains contained imide derivatives, and analogues **10i**–**k**, **11l**–**n**, **12o**–**p**, and **16q**–**s** exhibited weak inhibitory effects against the Hep3B cell line but displayed very low effects against the HL-60, MCF7, and MDA-MB-231 cell lines. 

**Table 2:** Cytotoxicity effect of **1**, cantharidin-sulfanilamides (**5a**–**h**), and imide analogues (**10i**–**k**, **11l**–**n**, **12o**–**p**, and **16q**–**s**) in human hepatocellular carcinoma (Hep3B), myeloid leukemia (HL-60), and human breast adenocarcinoma (MCF7 and MDA-MB-231) cell lines.

Cpd No.	IC_50_ (μM) ^a^			
	HL-60	Hep3B	MCF7	MDA-MB-231
**1**	5.6	3.4	-	-
**5a**	38.2	9	>100	>100
**5b**	>100	>100	>100	>100
**5c**	>100	>100	>100	>100
**5d**	>100	>100	>100	>100
**5e**	>100	>100	>100	>100
**5f**	>100	>100	>100	>100
**5g**	>100	17	>100	>100
**5h**	>100	80	>100	>100
**10i**	>100	>100	>100	>100
**10j**	>100	>100	>100	>100
**10k**	>100	>100	>100	>100
**11l**	>100	>100	>100	>100
**11m**	>100	>100	>100	>100
**11n**	>100	>100	>100	>100
**12o**	>100	>100	>100	>100
**12p**	>100	>100	>100	>100
**16q**	>100	>100	>100	>100
**16r**	>100	>100	>100	>100
**16s**	>100	>100	>100	>100

^a^ IC_50_ was calculated after 48 h of continuous drug exposure, values are the mean of three to four experiments with coefficients of variation of 5%–10%.

The cytotoxicity of cantharidin was investigated in both the NCI and Oncotest cell line panels [37]. It showed profound activity at low micromolar concentrations (log_10_IC_50_ values of −6.980 to −5.009 M), and revealed extreme cytotoxicity toward cell lines of diverse tumor types. According to a COMPARE analysis [38] of microarray-based transcriptome-wide mRNA expressions, 21 genes were found to be significantly correlated with the response of 60 tumor cell lines to cantharidin. Some researchers screened their synthetic compounds against human cancer cell lines and used a COMPARE analysis to evaluate the anticancer activity and predict the probable mechanism [27,39,40,41,42]. Therefore, the following studies examined the possible mechanism using a standard NCI-60 test and COMPARE analysis of **5a**.

## 3. Experimental Section

### 3.1 Synthesis

#### 3.1.1. General Experimental Procedures

General procedures were followed for the reaction of ammonia with cantharidine and the other acid anhydride. These compounds were prepared according to similar procedures, and the reactions took place in high-pressure tubes (Büchi Glasuster 0032, Zürich, Switzerland). Cantharidin or acid anhydrides were added to a tube containing 3 mL of dry toluene and triethylamine (TEA), and the solution was stirred and heated to *ca.* 200 °C. After being stirred for 2 h, the mixture was evaporated, and the residual mass was purified by column chromatography on silica gel and recrystallized from methanol.

All temperatures are reported in degrees centigrade. Melting points were determined with a Büchi B-545 melting point apparatus (Büchi, Flawil, Switzerland) and were uncorrected. ${\left[\alpha \right]}_{D}^{20}$ values were recorded on a JASCO P-1020 Digital Polarimeter (JASCO, Tokyo, Japan). Infrared spectra were recorded on a Perkin-Elmer Model 882 (Thermo Fisher Scientific Inc., New York, NY, USA) and Nicolet 510 pet and Thermo Mattson IR 300 (Thermo Fisher Scientific Inc.) spectrophotometers. ^1^H-nuclear magnetic resonance (NMR) spectra (in CDCl_3_ unless otherwise stated) were recorded at 500 MHz on a Bruker Advance DRX and AM-500 FT (Bruker Co., Bremen, Germany). Mass spectra were obtained on a JOEL JMSHX 110 FAB-MS (Joel, Tokyo, Japan) spectrometer and MAT-95XL HRMS (Finnigan, München, Germany).

#### 3.1.2. Test Samples

Chinese blister beetles were extracted with a water–ethanol (1:1) solution, filtered through celite, purified by chromatography on silica gel, and then recrystallized with ethanol to give cantharidin **1**. Compounds **5a**–**h**, **10i**–**k**, **11l**–**n**, **12o**–**p**, and **16q**–**s** were prepared from cantharidin, acid anhydride, and primary amines in the presence of TEA in toluene in high-pressure tubes. The mass spectra of all compounds were measured, and ^1^H-NMR was also used for testing.

#### 3.1.3. Physical Data of Cantharidinimide Derivatives and Analogues

*N-Cantharidinimido-sulfamethazine* (**5a**): 62% yield, m.p. 195–196 °C. IR (KBr): 1709 (amide) cm^−1^; ^1^H-NMR: δ 1.25 (6H, s), 1.75–1.77 (2H, m), 1.86–1.87 (2H, m), 2.05 (6H, s), 4.69 (2H, t, *J* = 2.3, 2.6 Hz), 6.64 (1H, s, pyrimidinyl H), 7.52 (2H, d, *J* = 8.6 Hz, phenyl H), 8.26 (2H, d, *J* = 8.7 Hz, phenyl H); FAB-MS *m/z* (rel. int.): 457 [M + H]^+^ (100), 154, 124; HRMS, calcd. for C_22_H_25_N_4_O_5_S [M + H]^+^ 457.1546, found 457.1547.

*N-Cantharidinimido-sulfisomidine* (**5b**): 49% yield, m.p. 163–164 °C. IR (KBr): 1710 (amide) cm^−1^; ^1^H-NMR: δ 1.26 (6H, s), 1.75–1.77 (2H, m), 1.78–1.80 (2H, m), 2.54 (3H, s), 2.67 (3H, s), 4.69 (2H, t, *J* = 2.3 Hz), 6.9 (1H, s, pyrimidinyl H), 7.55 (2H, d, *J* = 8.4 Hz, phenyl H), 8.06 (2H, d, *J* = 8.4 Hz, phenyl H); FAB-MS *m/z* (rel. int.) 457 [M + H]^+^ (22), 279, 154; HRMS (FAB^+^), calcd. for C_20_H_22_N_3_O_6_S 457.1546, found 457.1549.

*N-Cantharidinimido-sulfamerazine* (**5c**): 10% yield, m.p. 254–256 °C. IR (KBr): 1710 (amide) cm^−1^; ^1^H-NMR: δ 1.25 (6H, s), 1.74–1.76 (2H, m), 1.84–1.87 (2H, m), 2.42 (3H, s), 4.68 (2H, t, *J* = 2.3 Hz), 6.80 (1H, d, *J* = 5.2 Hz, pyrimidinyl H), 7.52 (2H, d, *J* = 8.5 Hz, phenyl H), 8.24 (2H, d, *J* = 8.6 Hz, phenyl H), 8.42 (1H, d, *J* = 5.1 Hz, pyrimidinyl H); HRMS (FAB^+^), calcd for C_21_H_23_N_4_O_5_S 443.1311, found 443.1313.

*N-Cantharidinimido-sulfaquinoxaline* (**5d**): 6% yield, m.p. 253–255 °C. IR (KBr):1704 (amide) cm^−1^; ^1^H-NMR: δ 1.20 (6H, s), 1.67–1.71 (2H, m), 1.80–1.84 (2H, m), 4.63 (2H, t, *J* = 2.8, 2.4 Hz), 7.45–7.48 (3H, m, H-6’ peak overlap, *J* = 8.5 Hz), 7.58 (2H, t-like, 2H), 7.89 (1H, d, *J* = 8.2 Hz, H-8’), 8.14 (2H, d, *J* = 8.6 Hz), 8.59 (1H, s). HRMS (FAB^+^), calcd. for C_24_H_22_N_4_O_5_S 478.1305, found 478.1307.

*N-Cantharidinimido-sulfacarbamide* (**5e**): 2% yield, m.p. 115–116 °C. IR (KBr): 1776, 1706 (amide) cm^−1^; ^1^H-NMR (DMSO-*d*_6_): δ 1.28 (6H, s), 1.69–1.70 (2H, m), 1.95–1.96 (2H, m), 4.63 (2H, t-like), 7.54 (2H, d, *J* = 8.6 Hz), 8.02 (2H, d, *J* = 8.4 Hz); HRMS, calcd. for C_16_H_18_N_2_O_5_S 350.0894, found 350.0895.

*N-Cantharidinimido-N’-(2-thiazolyl)-sulfanilamide* (**5f**): 10% yield, m.p. 125–127 °C. IR (KBr): 1706 (amide) cm^−1^; ^1^H-NMR: δ 1.25 (6H, s), 1.74–1.76 (2H, m), 1.85-1.87 (2H, m), 4.68 (2H, s), 6.54 (1H, d, *J* = 4.5 Hz, thiazolyl N-CH), 7.12 (1H, d, *J* = 4.5 Hz, thiazolyl S-CH), 7.47 (2H, d, *J* = 8.2 Hz, phenyl H), 8.02 (2H, d, *J* = 8.5 Hz, phenyl H); FAB-MS m/z (rel, int): 434 [M + H]^+^ (50), 154 (100); HRMS, calcd. for C_19_H_20_N_3_O_5_S_2_ 434.0844, found 434.0847.

*N-Cantharidinimido-sulfamethoxazole* (**5g**): 35% yield; m.p. 211–213 °C. IR (KBr): 1710 (C=O) cm^−1^; ^1^H-NMR (acetone-*d*_6_): δ 1.58 (6H, s), 2.25–2.26 (4H, m), 5.02 (2H, t, *J* = 2.8 Hz), 2.78 (3H, s), 6.69 (1H, s), 8.01 (2H, t, *J* = 6.9 Hz), 8.48 (2H, d, *J* = 6.7, 1.9 Hz); FAB-MS *m/z* (rel, int): 432 [M + H]^+^ (100); HRMS calcd. for C_20_H_22_N_3_O_6_S 432.1229, found 432.1226.

*N-Cantharidinimido-sulfadiazine* (**5h**): 27% yield; 262–263 °C; IR (KBr): 1710 (amide) cm^−1^; ^1^H-NMR (acetone-*d*_6_): δ 1.27 (6H, s), 1.64–1.66 (4H, m), 4.57 (2H, t, *J* = 2.8 Hz), 7.06 (1H, t, *J* = 4.7 Hz), 7.54 (2H, t, *J* = 4.3 Hz), 8.23 (2H, t, *J* = 4.3 Hz), 8.50 (2H, d, *J* = 5.0 Hz); FAB-MS *m*/*z* (rel, int) 429 [M + H]^+^ (100); HRMS calcd. for C_20_H_21_N_4_O_5_S 429.1233, found 429.1229.

*N-(2,2-Dimethylethyl-p-toluolsulfonamide)cantharidinimide* (**10i**): 14% yield, 154–155 °C. IR (KBr): 1147 (S(=O)_2_), 1401 (S(=O)_2_), 1691 (C=O), 3264 (NH) cm^−1^; ^1^H-NMR (see Table 1); HRMS, calcd. for C_21_H_28_N_2_O_5_S 420.1719, found 420.1718.

*N-(2-Methylethyl-p-toluolsulfonamide)cantharidinimide* (**10j**); 21% yield, m.p. 223–225 °C. IR (KBr): 1139 (S(=O)_2_), 1397 (S(=O)_2_), 1691 (C=O), 3244 (NH) cm^−1^; ${\left[\alpha \right]}_{D}^{20}$ −5.6 (*c* 0.5, CHCl_3_); ^1^H-NMR (see Table 1); HRMS, calcd. for C_20_H_26_N_2_O_5_S 406.1562, found 406.1560.

*N-(2,2-Dimethylethyl-benzosulfonamide)cantharidinimide* (**10k**): 22% yield, m.p. 155-156 °C. IR (KBr): 1152 (S(=O)_2_), 1400 (S(=O)_2_), 1695 (C=O), 3253 (NH) cm^−1^; ^1^H-NMR: δ 1.13 (6H, s), 1.23 (6H, s), 1.68–1.70 (2H, m), 1.78–1.81 (2H, m), 3.52 (2H, s), 4.53 (2H, t, *J* = 2.4 Hz), 5.88 (1H, s), 7.44 (2H, t, *J* = 7.4 Hz), 7.49 (1H, t, *J* = 7.3, 7.2 Hz), 7.84 (2H, d, *J* = 7.5 Hz); HRMS, calcd. for C_20_H_26_N_2_O_5_S 406.1562, found 406.1564.

*N-[2,2-Dimethylethylamino]-1-naphtharinimido-4-methylsulfanilamide* (**11l**): 10% yield, m.p. 247–248 °C (MeOH). IR (KBr): 1151 (S(=O)_2_), 1384 (S(=O)_2_), 1715 (C=O), 3284 cm^−1^; ^1^H-NMR (CDCl_3_, 500 Hz): δ 1.28 (6H, s), 2.30 (3H, s), 3.89 (2H, s), 5.69 (1H, s), 7.10 (2H, d, *J* = 8.1 Hz), 7.67 (2H, d, *J* = 8.2 Hz), 7.78 (2H, dt-like, *J* = 4.0, 7.0, 8.5 Hz), 7.88 (2H, dt-like, *J* = 3.1, 5.3, 6.8 Hz); HRMS (EI, 80 ev), calcd. C_19_H_20_N_2_O_4_S 372.1144, found 372.1143.

*N-[2-Methylethylamino]-1-naphtharinimido-4-methylsulfanilamide* (**11m**): 73% yield, m.p. 200–202 °C (MeOH). IR (KBr): 1135 (S(=O)_2_), 1397 (S(=O)_2_), 1711 (C=O), 3313 cm^−1^; ^1^H-NMR: *δ* 1.28 (3H, d, *J* = 6.4 Hz), 2.09 (3H, s), 3.52 (2H, dt-like, *J* = 4.5, 10.1, 14.4 Hz), 3.68 (1H, d, *J* = 7.6 Hz), 4.88 (1H, d, *J* = 7.9 Hz), 6.86 (2H, d, *J* = 8.0 Hz), 7.53 (2H, d, *J* = 8.1 Hz), 7.71 (4H, dt-like, *J* = 5.4, 9.0, 10.2 Hz); HRMS (EI, 80 ev), calcd. C_18_H_18_N_2_O_4_S 358.0987, found 358.0986.

*N-[2,2-Dimethylethylamino]-1-naphtharinimidosulfanilamide* (**11n**): 34% yield, m.p. 202–203 °C (MeOH). IR (KBr): 1155 (S(=O)_2_), 1392 (S(=O)_2_), 1715 (C=O), 3276 cm^−1^; ^1^H-NMR: δ 1.29 (6H, s), 3.67 (2H, s), 5.78 (1H, s), 7.31 (2H, t, *J* = 7.7 Hz), 7.39 (1H, t, *J* = 7.5 Hz), 7.76 (2H, qd, *J* = 7.5, 5.5, 3.1, 3.1 Hz), 7.81 (2H, dd-like *J* = 7.7, 1.1), 7.88 (2H, dd, *J* = 3.1, 5.4 Hz); HRMS (EI, 80 ev), calcd. C_18_H_18_N_2_O_4_S 358.0987, found 358.0989.

*N-[3-(1,3-Dioxo-1,3,3a,4,7,7a-hexahydro-isoindol-2-yl)-2,2-dimethylethyl]-4-methyl-benzenesulfonamide* (**12o**): 22% yield, m.p. 133–134 °C (MeOH). IR (KBr): 1143 (S(=O)_2_), 1397 (S(=O)_2_), 1707 (C=O), 3272 (NH) cm^−1^; ^1^H-NMR: δ 1.20 (6H, s), 2.22 (2H, dd, *J* = 4.2, 4.6 Hz), 2.40 (3H, s), 2.57 (2H, dd, *J* = 1.6, 1.6 Hz), 3.10 (2H, t, *J* = 2.8 Hz), 3.47 (2H, s), 5.79 (2H, t, *J* = 3.1Hz), 5.85 (1H, s), 7.25 (2H, d, *J* = 9.4 Hz), 7.71 (2H, d, *J* = 8.2 Hz); HRMS (EI, 80 ev), calcd. C_19_H_24_N_2_O_4_S 376.1457, found 376.1458.

*N-[3-(1,3-Dioxo-1,3,3a,4,7,7a-hexahydro-isoindol-2-yl)-2-methylethyl]-4-methyl-benzenesulfonamide* (**12p**): 22% yield, m.p. 145–146 °C (MeOH). IR (KBr): 1160 (S(=O)_2_), 1401 (S(=O)_2_), 1695 (C=O), 3280 (NH) cm^−1^; ^1^H-NMR: δ 1.00 (3H, d, *J* = 6.6 Hz), 2.23 (2H, dd, *J* = 5.3, 15.5 Hz), 2.41 (3H, s), 2.54 (2H, d, *J* = 18.1 Hz), 2.96 (1H, dd, *J* =2.7, 2.9 Hz), 3.01 (1H, dd, *J* = 2.7, 10.7 Hz), 3.31 (1H, dd, *J* = 3.7, 13.7 Hz), 3.49 (1H, dt-like, *J* = 5.9, 9.4, 10.9 Hz), 3.61 (1H, s), 4.78 (1H, d, *J* = 8.4 Hz), 5.88 (2H, t, *J* = 2.2 Hz), 7.28 (2H, d, *J* = 8.1 Hz), 7.70 (2H, d, *J* = 8.1 Hz); HRMS (EI, 80 ev), calcd. for C_18_H_22_N_2_O_4_S 362.1300, found 362.1298.

*exo-N-Hydroxyl-7-oxabicyclo[2.2.1]hept-5-ene-2-phenylsulfonamido-1-methylethyl-2,3-dicarboximide* (**16q**): 13% yield, m.p. 127–133 °C (MeOH). IR (KBr): 1709 (C=O) cm^−1^; ^1^H-NMR: δ 1.34 (3H, d, *J* = 3.3 Hz), 2.71 (2H, s), 2.99–3.03 (2H, m, N-CH_2_), 4.23–4.24 (1H, m, O-CH), 5.02 (1H, s), 5.19 (1H, s), 6.47 (2H, s), 7.49–7.55 (3H, m), 7.85–7.86 (2H, m); FAB-MS *m/z* (rel. int. %): 379 [M + H]^+^ (15), 311 (48), 270 (34), 70 (75), 56 (100).

*exo-N-Hydroxyl-7-oxabicyclo[2.2.1]hept-5-ene-2-phenylsulfonamido-2-methylethyl-2,3-dicarboximide* (**16r**): 9% yield; 174–176 °C (MeOH); IR (KBr): 1690 (C=O) 3327 cm^−1^; ^1^H-NMR: δ 1.23 (3H, d, *J* = 2.8 Hz), 2.74 (2H, dd, *J* = 5.4, 7.3 Hz), 3.88 (2H, td, *J* = 2.8, 4.2, 8.2 Hz), 3.51 (1H, dd, *J* = 3.1, 4.4 Hz), 5.24 (2H, d, *J* = 4.9 Hz), 6.50 (2H, s), 7.49 (3H, dt-like, *J* = 5.8, 6.0, 11.3 Hz), 7.84 (2H, dt-like, *J* = 5.4, 6.0, 9.9 Hz); FAB-MS *m/z* (rel. int. %): 379 [M + H]^+^ (3), 135 (70), 74 (75), 197 (35).

*exo-N-Hydroxyl-7-oxabicyclo[2.2.1]hept-5-ene-2-[(2’,5’-dichlorophenyl)sulfonamidoethyl]-2,3-dicarboximide* (**16s**): 48% yield, m.p. 212-216 °C (MeOH). IR (KBr): 1780 cm^−1^; ^1^H-NMR: *δ* 2.77 (2H, s), 3.21 (2H, dd, *J* = 5.5, 10.1 Hz), 4.09 (2H, t, *J* = 9.5 Hz), 5.25 (2H, s), 6.50 (2H, s), 6.40 (1H, t, 11.9 Hz), 7.44 (2H, s), 8.04 (1H, s); FAB-MS *m/z* (rel. int. %): 433 [M + H]^+^ (4), 155 (30), 365 (10); HRMS (FAB^+^), calcd. for C_16_H_15_N_2_O_6_Cl_2_S 433.0029, found 433.0005.

### 3.2. Antineoplastic Bioassays

#### 3.2.1. Cell Culture

Media and sera for cell culture were purchased from Gibco/BRL (Grand Island, NY, USA). Most chemicals were purchased from Sigma Chemical (St. Louis, MO, USA). The human hepatoma (Hep3B) and myeloid leukemia (HL-60) cell lines were obtained from the American Type Culture Collection (ATCC; Rockville, MD, USA). The human breast cancer cell lines, MDA-MB-231 and MCF-7, were purchased from the cell bank of the National Health Research Institute (Miaoli, Taiwan). These cells were maintained as monolayers in Dulbecco’s modified Eagle’s medium (DMEM) containing 10% heat-inactivated fetal bovine serum (FBS), 100 units/mL penicillin, 100 μg/mL streptomycin, 100 μM nonessential amino acids, and 1 mM glutamine in a controlled atmosphere of 5% CO_2_ and 95% air at 37 °C.

#### 3.2.2. MTT Assay for Cellular Viability

Cells were seeded into 96-well plates and allowed to adhere for 24 h before drugs were introduced. Following a 48-h incubation, drugs and media were removed, and each well was treated with 100 μL of 500 μg/mL 3-(4,5-dimethylthiazol-2-yl)-2,5-diphenyltetrazolium bromide (MTT) in culture medium. Following a 4-h incubation period to allow the metabolism of MTT by mitochondrial dehydrogenases of viable cells to form an insoluble formazan product, the plates were centrifuged at 450× *g* for 10 min, and supernatants were removed and replaced with 100 μL DMSO. The plates were shaken to maximize solubilization of the formazan crystals. The absorbance, as a measure of viable cell numbers, was read the following day in a model MA310 automated EIA plate reader (Whittaker M. A. Bioproducts, Inc., Walkersville, MD, USA) at a wavelength of 550 nm. IC_50_ values were obtained by a linear regression analysis of the percent absorbance *vs.* the log of the drug concentration. It was previously shown that viable cell numbers are correlated with the optical density as determined in the MTT assay.

## 4. Conclusions

We synthesized cantharidinimide derivatives, **5a**–**h**, and their analogues **10i**–**k**, **11l**–**n**, **12o**–**p**, and **16q**–**s**. The most potent cytostatic compound, *N*-cantharidinimido-sulfamethazine (**5a**), exhibited anti-HL-60 and anti-Hep3B cell activities. Two compounds, **5g** and **5h**, displayed slight effects on the Hep3B cell line, while the other compounds produced no response in these four cell lines. Although they exhibited very low effects on human carcinoma cells, we observed that these compounds contained sulfamoyl moieties. When studying the structure-activity relationship, we found that these compounds had very similar chemical structures to some diuretics, antidiabetic drugs, and hypoglycemic agents. Thus, their pharmacological effects should be tested in the future.
